# Systemic involvement in ACS: Using CMR imaging to compare the aortic wall in patients with and without acute coronary syndrome

**DOI:** 10.1371/journal.pone.0203514

**Published:** 2018-12-12

**Authors:** Elizabeth Chandy, Alexander Ivanov, Devindra S. Dabiesingh, Alexandra Grossman, Prasanthi Sunkesula, Lakshmi Velagapudi, Virna L. Sales, Edward J. Colombo, Igor Klem, Terrence J. Sacchi, John F. Heitner

**Affiliations:** 1 Division of Cardiology, Institute for Cardiology and Cardiac Surgery, NewYork-Presbyterian Brooklyn Methodist Hospital, Brooklyn, New York, United States of America; 2 Division of Cardiology, Department of Medicine, Duke University Medical Center, Durham, North Carolina, United States of America; Centro Cardiologico Monzino, ITALY

## Abstract

**Background/Objectives:**

Previous studies have demonstrated that in acute coronary syndrome (ACS), plaque destabilization and vessel inflammation, represented by vessel edema, often occur simultaneously in multiple coronaries, as well as extend to the cerebrovascular system. Our aim was to determine whether the inflammatory vascular processes occurring within the coronaries during ACS extend simultaneously to the descending aorta.

**Methods:**

We prospectively enrolled 111 patients (56 ACS patients and 55 non-ACS patients with known coronary artery disease) to undergo cardiac magnetic resonance of the thoracic aortic wall at presentation and at three-month follow-up. The primary outcome was change in aortic wall area (AWA) and maximal aortic wall thickness (AWT) from baseline to three-month follow-up. Secondary outcomes were baseline and follow-up differences in AWA and AWT, and changes in C-reactive protein (CRP).

**Results:**

There was a significant reduction in mean AWA (p = 0.01) and AWT (p = 0.01) between index and follow up scans in ACS group, with no significant changes in non ACS group (both p>0.1) and no difference between ACS and non-ACS groups (p = 0.22). There was no significant difference in AWA and AWT at baseline (p>0.36) and follow-up (p>0.2) between groups. There was a significant reduction in CRP in both groups (p<0.01), with higher reduction in ACS patients (p<0.01)

**Conclusions:**

There was a reduction in aortic wall size, aortic wall area, and aortic wall thickness in patients presenting with ACS, and no change in non-ACS patients. There were no interval between-group differences in these measurements. We observed a reduction in C-reactive protein in both groups, with higher reduction noted in ACS patients.

## Introduction

Acute coronary syndrome (ACS) accounts for nearly half of cardiovascular disease morbidity and mortality worldwide and is usually the direct result of atherosclerotic plaque instability [1, 2]. Although plaque instability is typically transient and challenging to reference with noninvasive imaging, associated concurrent or lingering vessel wall edema from endothelial inflammation can act as a marker for this occurrence [3, 4, 5]. Beyond directly reflecting the inciting coronary, features of mural edema is not just a reflection on coronary events but occurs elsewhere as a systemic response during ACS [6, 7].

Studies have shown that patients with ACS often present with evidence of vessel injury in multiple coronary arteries rather than of a single coronary vessel [6, 7]. In fact, beyond the coronary system, it is postulated that large vessel arterial systems are also simultaneously involved in the inflammatory process of ACS. In both animal and human models, increased numbers of unstable plaques were noted on carotid evaluations during ACS, translating, in some cases, to co-incident ischemic strokes [8, 9]. Likewise, increased complex morphologies of plaque burden, including edema within the abdominal aorta wall, were found in ACS patients as compared to those with stable coronary artery disease (CAD) [10]. Furthermore, a prior cardiac magnetic resonance (CMR) study has demonstrated a greater thoracic aortic wall thickness, as a surrogate measure of inflammatory mural edema, in patients presenting with ACS compared to diabetic patients without ACS [11, 12].

Therefore, this study was designed to assess whether the inflammatory process of ACS occurs throughout the vascular system via assessment of the thoracic aortic wall edema. Utilizing CMR imaging’s high-resolution capabilities of visualizing aortic vascular wall tissue, at index presentation, measurements of the aortic wall area (AWA) and maximal aortic wall thickness (AWT) of ACS patients were compared to patients with stable CAD. A three-month follow-up assessment of the same aortic wall by CMR was then performed to look for regression of aortic wall mural edema.

## Methods

This prospective cohort study was approved by NewYork Presbyterian Brooklyn Methodist Hospital institutional research ethics board and each study participant signed a written informed consent for study enrollment.

### Study population

We prospectively enrolled patients admitted to a Brooklyn, New York community teaching hospital with an acute myocardial infarction, either non-ST elevation or ST elevation, between April 2012 and November 2014. Patients underwent imaging by CMR of their descending thoracic aortic wall during their hospital stay. These patients were compared to a non-ACS group, consisting of patients presenting for outpatient elective coronary angiogram who did not have ACS (chest pain at rest) but had established obstructive CAD by visual angiographic criteria (≥70% stenosis in any coronary vessel or ≥50% stenosis of left main vessel) on cardiac catheterization. Myocardial infarction was defined according to 2007 ACC/AHA guidelines as acute chest discomfort or angina equivalent with associated elevated troponin level over 99% of the normal reference [13]. Excluded patients were those who were less than 18 years of age; hemodynamically unstable (heart rate >100 per minute or systolic blood pressure <90 mmHg); pregnant; had an active infectious process (fever >100.4°F, positive cultures or anti-microbial treatment), active malignancy, or prior myocardial infarction within 90 days; or with a contraindication to MRI (implanted pacemaker or defibrillator, aneurysmal clips, recent metallic implant, claustrophobia, or body habitus not permissible with the imaging equipment). Baseline biographic data were obtained at the time of CMR from an itemized questionnaire and chart review. To decrease potential selection biases recruitment was simultaneous in both groups.

### Magnetic resonance image protocol

All patients were imaged using a 1.5-T CMR system (Siemens Healthcare, MAGENTOM-Avanto) with a six-channel phased-array receiver coil. After localization with a fast gradient echo sequence, a single slice, ECG retro-gatedT-2 weighted turbo spin echo, dark blood saturated imaging with fat saturation was obtained with the following parameters: pixel size 1.0 x 1.0 mm, slice thickness of 6.0 mm, field of view in the read direction was 200 mm with 50% over-sampling, 100% field of view in the phase direction, TR 1600 msec, and TE 69 msec. ACS patients were imaged within 72 hours of diagnosis and non-ACS patients were imaged on the day of their outpatient catheterization procedure. Both groups were re-imaged at three-month follow-up (90 ± 10 days).

### Analysis of aortic wall

**Fig 1:**
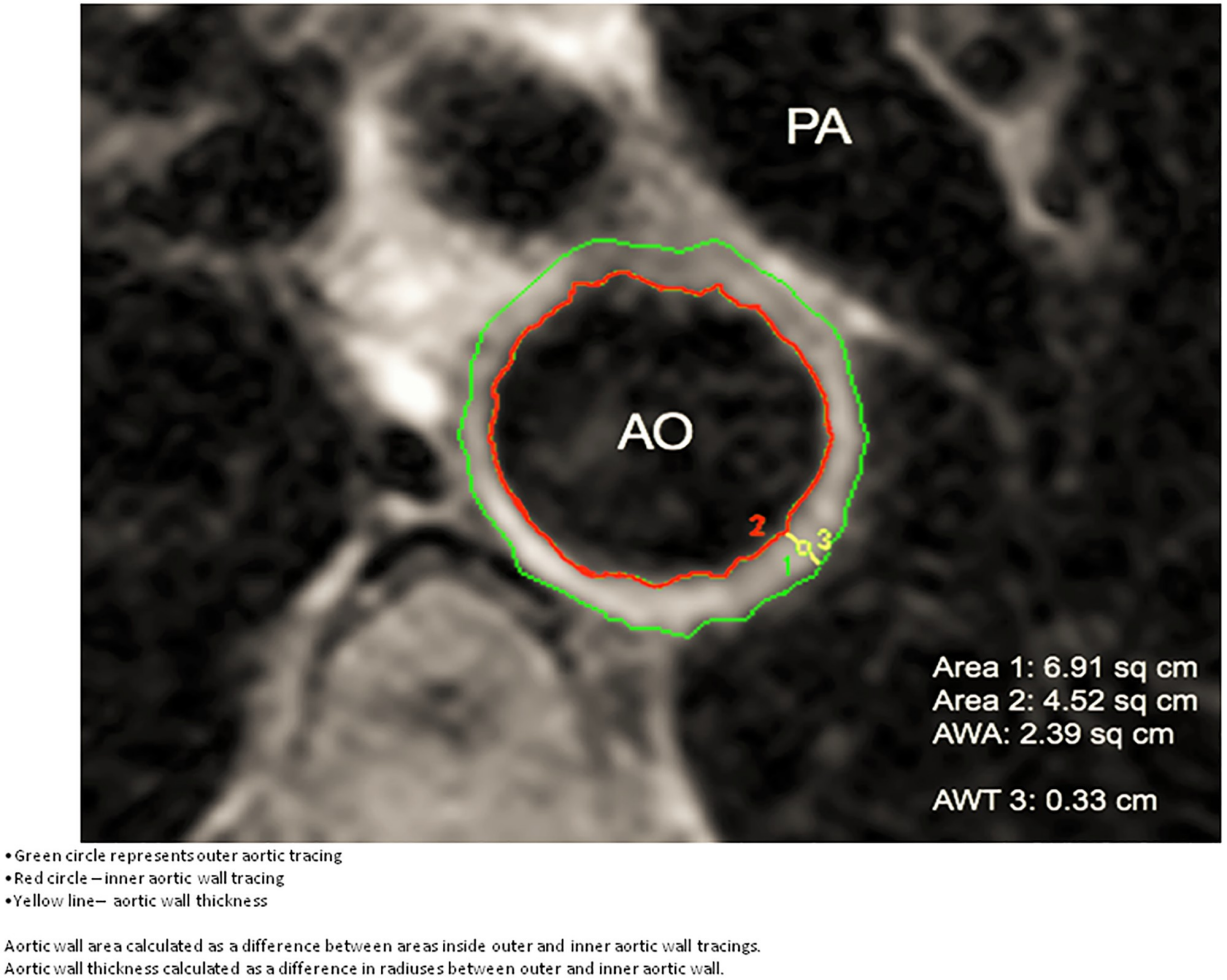
T1 weighted image of a descending aorta at the level of pulmonary artery bifurcation.

Images were obtained perpendicular to two long axis views of the descending thoracic aorta at the level of the proximal bifurcation of the pulmonary artery in each patient. Once the aortic wall image was obtained on the first visit, a standard assessment was applied to the acquired images by measuring the distance from the proximal end of the left subclavian artery as a fixed landmark and applying this distance for the follow-up aortic wall measurement. Wall assessment was performed with computer-assisted morphometric analysis (Siemens, Argus work station) of the cross sectional image after two region of interest (ROI) curves (an inner luminal curve and an outer tracing curve) was obtained by two SCMR level II competent observers blinded to the patient clinical data [14]. There were 2 interpreters that performed the analysis. Both interpreters performed the same measurements on 20 patients to assess for inter-observer variability. The interpreters were blinded to all patient information, including whether the study was a baseline or follow-up study. AWA was calculated in centimeters squared between the inner and outer ROI curves [11,15,16]. AWT in centimeters was measured at the thickest portion of the aortic wall (Fig 1).

### Aortic wall measurement by CMR. ROI measurements of the descending thoracic aortic wall

The difference in area generated by the outer tracing curve (1, green) and the inner luminal curve (2, red) is the aortic wall area. Maximal aortic wall thickness is taken at the largest perpendicular distance (3, yellow) between the two drawn curves. AO = aorta, PA = main pulmonary artery(Fig 1).

### Serum C-reactive protein (CRP) measurement and biomarker collection

Blood draws for hs-CRP (high sensitivity CRP) levels were taken within 48 hours of diagnosis for the ACS group and at enrollment for the non-ACS group. Serum lab results for cardiac ischemic biomarkers (troponin I, creatinine kinase isozyme-MB [CKMB]), lipid markers (total cholesterol, triglycerides, high density lipoprotein [HDL] and low density lipoprotein [LDL]), and creatinine level were also collected. At three months (90 ± 10 days) after ACS or non-ACS enrollment, blood draws were again taken for follow-up analysis.

### Outcomes

Primary outcome was the change in AWA and AWT from baseline to the three-month follow-up, between ACS and non-ACS patients. Secondary outcomes examined for differences in AWA and AWT within groups at baseline and three-month follow-up, as well as, changes in serum hs-CRP inflammatory marker levels.

### Statistical methods

Categorical variables were reported as proportions and continuous variables with normal distribution were reported as mean ± standard deviation, continuous variables with abnormal distribution were presented as median (interquartile range)We used analysis of variance test for paired observation to calculate the significance of changes over time. Additionally we used multivariate linear regression to compare measurements at follow up adjusted for baseline level of the corresponding measurements, age, gender, BMI and statin therapy at follow up (for lipids and CRP only). To identify these variables we identified variables associated with outcome in a univariate regression and then used forward and backward method to identify the most informative model using AKI. Given our sample size we included up to 5 controlling variables in the tested models. Two-sided p-values were used and a probability value of less than 0.05 was indicative of statistical significance. All analysis was performed using Stata 14.2 (StataCorp, Texas USA).

## Results

### Enrollment data

One hundred eleven patients (56 ACS and 55 non-ACS) were included for final CMR imaging and analysis. Twelve patients (11 presenting with ACS and 1 in the non ACS group) had to be excluded due to poor quality CMR images. There was no statistical difference for age or body mass index between the groups. There was a trend towards larger prevalence of males. Greater than 80% of the enrolled females were postmenopausal. Higher prevalence of prior CAD, myocardial infarction, percutaneous coronary intervention, coronary artery bypass grafting, diabetes mellitus, hypertension and dyslipidemia were noted in the non-ACS group. Differences in medication use between the groups were noted as well (Table 1 and S1 Table). Of interest there was a significant difference in number of patients on statin therapy at the baseline between ACS and non-ACS group (88% vs 46%, p<0.001). At the follow-up there was a significant increase in number of patients taking statin in ACS group (to 88%, p<0.001), with 97% (p>0.3 for increase) of the patients in non-ACS group being on statin therapy, and no difference between groups at follow-up (p>0.93). In the ACS group, mean peak troponin level was 22.5 ± 37.2 ng/mL and mean peak CKMB level was 66.5 ± 107.6 ng/mL. Complete baseline characteristics are presented in Table 1, S1 Table.

**Table 1:** Baseline characteristics and three-month follow-up of medication use.

	Enrollment				
	ACS n = 56	[%]	Non-ACS n = 55	[%]	*p*-Value
Age (years)	60 ± 13		62 ± 9		0.41
Male	36	[64%]	40	[73%]	0.42
BMI (%)	29 ± 6		29 ±5		0.75
Medical History					
CAD	15	[27%]	55	[100%]	<0.001
Myocardial Infarction	6	[11%]	14	[25%]	0.051
PCI	14	[25%]	45	[82%]	<0.001
CABG	1	[2%]	9	[16%]	0.008
Diabetes Mellitus	16	[28%]	29	[53%]	0.012
Hypertension	32	[57%]	52	[94%]	<0.001
Dyslipidemia	32	[57%]	51	[93%]	<0.001
Peripheral Artery Disease	1	[2%]	2	[4%]	0.62
TIA or Ischemic Stroke	3	[5%]	5	[9%]	0.49
Family History of CAD	23	[41%]	18	[33%]	0.43
History of Smoking	25	[45%]	29	[53%]	0.45
Postmenopausal Females	17	[85%]	14	[93%]	0.62
Medication Use					
Aspirin	53	[95%]	54	[98%]	0.62
P2Y12 Receptor Inhibitor	44	[79%]	52	[94%]	0.023
Statin	49	[87%]	53	[96%]	0.16
Beta Blocker	48	[85%]	47	[85%]	>0.99
Calcium Channel Blocker	7	[12%]	24	[44%]	<0.001
ACE Inhibitor	36	[64%]	28	[51%]	0.18
ARB	6	[11%]	8	[14%]	0.58
Diuretic	15	[27%]	20	[36%]	0.31
Peak Troponin (ng/mL)	7.9 (1.2; 28)				
Peak CKMB (ng/mL)	20.6 (8; 79)				
Peak Creatinine (mg/dL)	1 (0.9; 1.1)		0.9 (0.8; 1.1)		0.97
Total Cholesterol (mg/dL)	180 (147; 225)		153(128; 181)		<0.001
Triglycerides (mg/dL)	118 (75; 158)		132 (86; 157)		0.99
HLD (mg/dL)	41 (35; 55)		40(34; 49)		0.67
LDL (mg/dL)	115 (89; 152)		92 (71; 114)		<0.001
hs-CRP (mg/L)	21 (8; 47)		4 (1; 7)		<0.001

ACS = acute coronary syndrome; BMI = body mass index; CAD = coronary artery disease; PCI = percutaneous coronary intervention; CABG = coronary artery bypass grafting; TIA = transient ischemic attack; ACE = angiotensin converting enzyme; ARB = angiotensin II receptor blocker, CKMB = creatinine kinase isozyme-MB; HDL = high density lipoprotein; LDL = low density lipoprotein; hs-CRP = high sensitivity C-reactive protein.

Continuous variable presented as mean±SD (normal distribution) or median (interquartile range) (abnormal distribution)

### Aortic wall area and aortic wall thickness

Fifty-six ACS and 55 non-ACS patients underwent initial and three-month CMR analysis (Table 2). There was a significant reduction seen in AWA or AWT between baseline and three-month follow-up in the ACS group (p<0.01) with no significant differences in non-ACS group and between groups (p>0.22). The measured mean change of AWA from baseline to follow-up was -0.12cm^2^ (-0.3; 0.01) in the ACS group and -0.03 cm^2^ (-0.23; 0.11) in the non-ACS group; the mean change for AWT was -0.04 (-0.07; -0.01) and -0.01 cm (-0.05; 0.03)in the ACs and non ACS groups, respectively (all p>0.27 for difference between groups). Baseline AWA and AWT was 1.75 cm^2^ (1.55; 2.33) vs.1.83 cm^2^ (1.64; 2.24) and 0.33 cm (0.28; 0.41) vs. 0.34 cm (0.29; 0.42) in ACS vs. non-ACS patients (both p>0.5). There was no difference in follow-up measurements of AWA and AWT between the two groups 1.67cm^2^ (1.44; 2.24) vs. 1.79 cm^2^ (1.55; 2.15) and 0.32 cm (0.26; 0.38) vs. 0.32cm (0.27; 0.38) (both p>0.13) for ACS and non-ACS groups, respectively (S1 and S2 Figs).

**Table 2:** Aortic wall thickness and serum markers at enrollment versus at three-month follow-up.

Medication Use	ACS			Non-ACS			P value^1^
	Enrollment	Follow up	p value	Enrollment	Follow up	p value	
Aortic Wall Area (mm^2^)	1.75 (1.55;2.33)	1.67(1.44;2.34)	<0.005	1.83 (1.64;2.24)	1.79(1.55;2.15)	0.2	0.22
Aortic Wall Thickness (mm)	0.33 (0.28;0,41)	0.32 (0.26; 0.38)	<0.001	0.34 (0.29; 0.42)	0.34 (0.27; 0.38)	0.12	0.22
Total Cholesterol (mg/dL)	180 (147; 225)	153 (115; 188)	<0.001	153 (128; 181)	156 (138; 186)	0.17	<0.001
Triglycerides (mg/dL)	118 (75; 158)	125 (89; 193)	0.04	132 (86; 157)	138 (101; 206)	0.03	<0.005
HLD (mg/dL)	41 (35; 55)	42 (38; 49)	0.47	40(34; 49)	42 (36; 53)	0.17	0.82
LDL (mg/dL)	115 (89; 152)	89 (59; 103)	<0.001	92 (71; 114)	92 (74; 113)	0.5	<0.001
hs-CRP (mg/L)	21 (8; 47)	4 (1; 7)	<0.001	4 (1; 7)	2 (1; 4)	<0.003	<0.001

ACS = acute coronary syndrome; HDL = high density lipoprotein; LDL = low density lipoprotein; hs-CRP = high sensitivity C-reactive protein.

P value- comparing temporal changes within a group

P value^1^ –comparing temporal changes between groups

Continuous variable presented as (interquartile range).

### Serum markers

Higher total cholesterol (180mg/dL (147; 226) vs. 153mg/dL (128; 181), p <0.01) and higher LDL mg/dL (115 mg/dL (89; 152) vs.92mg/dL (71; 114), p <0.01) were demonstrated in the ACS patients at enrollment. There was a significant reduction in cholesterol and LDL level in the ACS group (at the follow-up in total cholesterol 153mg/dL (115; 188) and LDLmg/dL 89 (59; 103) (both p<0.01) with no difference in non ACS group 156mg/dL (138; 186) for cholesterol and 92 mg/dL (74; 113) for LDL (p>0.1). There was a very strong association between increased adherence to statin in ACS group and reduction in lipid markers (p<0.001). Hs-CRP levels at enrollment revealed notably higher levels in the ACS group (33 (9.5;37) mg/L) vs.4 (1.2;7.4) mg/L in the non-ACS group (p < 0.01). There was a strong association between increased adherence to statin in ACS group and reduction in lipid markers (p<0.001. Hs-CRP index level was higher in the ACS group (33 (9.5;37) mg/L) vs. 4 (1.2;7.4) mg/L in the non-ACS group (p < 0.01). There was a reduction in hs-CRP levels in both groups (p < 0.01) from index to follow up measurements, with larger reduction in ACS patients mg/L (4 (1.1; 6.8) vs. 1.9 mg/L (1; 3.2), p < 0.01) (Tables 1 and 2). Observed difference in CRP at baseline remained significant following adjustment (p<0.01) for age, gender, BMI, diabetes and therapy with statins at baseline. There was no difference between Hs- CRP levels at follow up after adjustment (p>0.52) with female gender, higher BMI, diabetes and baseline Hs-CRP being independent predictors.

### Inter-observer variability

Verification of no significant inter-observer variability was confirmed from a separate analysis of AWA and AWT measurements performed by another investigator. The inter-observer variability of aortic wall measurements was analyzed using the Bland-Altman method. All differences in measurements were within 2 SD of the mean. There was excellent intraclass correlation for all analyzed measurements with **Κ**>0.8.

## Discussion

This study found significant reduction in aortic wall area and maximal aortic wall thickness, inpatients presenting with ACS and no changes in non-ACS patients with known coronary artery disease with no significant difference between groups, at baseline compared to three-month follow-up. There was significant elevation in hs-CRP in the ACS group at baseline, and we observed a reduction in hs-CRP in both groups with a higher reduction in ACS patients.

The aim of this study was to identify if the inflammatory changes that occur in ACS extend to the systemic vasculature. It is well-known that cellular markers of inflammation, such as neutrophil myeloperoxidase, interleukin-6, and platelet-derived growth factor, are important factors in ACS, and their levels have also been shown to be elevated in remote vascular territories during ACS [17,18]. There is also evidence that often multiple coronaries, rather than a single coronary, are involved in the inflammatory process of ACS, suggesting a non-focal triggering of plaque rupture [6, 7]. In addition, prior studies utilizing carotid artery ultrasound demonstrated that complex, presumably unstable carotid plaques were much more prevalent in unstable angina patients compared to stable angina patients [8, 9]. Finally, an earlier one-time CMR comparison of 27 NSTEMI patients to non-cardiac chest pain and asymptomatic diabetic patients revealed both a greater AWA and AWT among the ACS patients on initial presentation. This study did not have follow-up images performed and thus could not definitely determine whether the finding was due to a higher burden of atherosclerosis versus a systemic involvement of the vascular system in the inflammatory process of ACS [11]. The changes in the AWA and AWT are postulated to be a result of both local and diffuse edema, lipid and macrophage insudation, chemokine-regulated vascular smooth muscle hypertrophy and deposition of microthrombi [2,19]. The clinical relevance of proposed acute, widespread, inflammation-driven plaque rupture includes a potential shift from the therapeutic strategies which focus on the specific plaque rupture to a more systemic therapeutic strategy, as well as potentially utilizing the easier accessible systemic vasculature for both diagnostic purposes as well surrogate endpoints for therapeutic strategies [20].

However, in this larger study, examining the theory that simultaneous aortic vascular inflammation occurs at the time of coronary vascular injury, and, as expected there was a significant reduction in AWA and AWT in ACS groups, with no difference in non ACS group and between ACS patients and non-ACS patients from baseline to three-month follow-up. Interestingly, there was no difference between groups at index and follow up scans. The lack of difference in AWA and AWT in the ACS vs non-ACS group is different from our previous study. A potential explanation might be related to the higher prevalence of risk factors for atherosclerosis in the non-ACS group and thus a higher atherosclerotic burden in the aorta. Interestingly we observed a reduction in CRP in not just the ACS group but in non-ACS patients as well. This suggests that reduction in AWA and AWT in these patients may be, at least partially, attributed to the reduction in inflammation. It is important to recognize that this study was designed to assess inflammation and not atherosclerosis via serial measurements of the wall area and thickness. A prior animal study showed regression of the inflammatory process of atherosclerosis, via reduction of macrophage foam cell deposition in aortic plaque, occurring as early as nine weeks after aggressive lipid-lowering intervention; in the same study, the histological findings well-correlated with the reduction of plaque thickness on MRI imaging [21]. Evaluation of the immunocytochemistry of carotid plaque of patients scheduled for carotid endarterectomy showed significantly less inflammation (fewer macrophages and T cells) with statin therapy after a time period of three months [22]. If the aortic wall area was involved in the inflammatory process of ACS, we would have expected to see a significant reduction in the three-month time frame.

Serum analysis for lipids and hs-CRP inflammatory markers aligned with typical constructs of ACS biology [23]. It is noted that with less prior treatment with statins, and recently destabilized coronary atherosclerotic plaque, the ACS patients had higher total cholesterol, LDL and hs-CRP levels. As markers are typically elevated in ACS patients, the significant reduction in LDL and hs-CRP observed at three-month follow-up in the ACS group was owed predominantly to the increased use of statins by follow-up (46% to 87%, p < 0.01). In fact, there was a notable increase in guideline recommended antiplatelet (50% to 95%, p < 0.01), and beta-blocker (50% to 85%, p < 0.01) use among the recovering ACS patients as well [24] (S1 Table). [25].

### Limitations

The predominant limitation is that only the thoracic aorta was assessed in this study to represent systemic vasculature, and other vascular areas, such as the abdominal aorta or carotids, should also be considered. Another limitation is the follow-up period of three months may not have been sufficient to demonstrate very gradual aortic wall edematous changes of the vascular healing process [25]. T1 weighted imaging was not performed for anatomical delineation, however we followed an originally described methodology for consistency purposes [11, 16].

## Conclusion

This study found a significant reduction in aortic wall area and maximal aortic wall thickness, in patients presenting with ACS with no difference in non-ACS patients with known coronary artery disease, at baseline compared to three-month follow-up and no between group differences, accompanied by reduction in C-reactive protein in both groups, with higher reduction notable in ACS patients.

## Supporting information

S1 Table a. Medication Use at Baseline and Three-Month Follow-Up. b. Temporal changes in Medication Use at Baseline and Three-Month Follow-Up.
(DOCX)

S1 Fig Aortic wall area. (DOCX)

S2 Fig Aortic wall thickness. (DOCX)

S3 Fig Examples of CMR changes in aortic wall area. (DOCX)
