# Supplementary material for: Systemic involvement in ACS: Using CMR imaging to compare the aortic wall in patients with and without acute coronary syndrome
Source: PLoS One. 2018 Dec 12;13(12):e0203514. doi: 10.1371/journal.pone.0203514 (PMC6291123; doi:10.1371/journal.pone.0203514)
Supplement: S1 Table — a. Medication Use at Baseline and Three-Month Follow-Up. b. Temporal changes in Medication Use at Baseline and Three-Month Follow-Up. (DOCX) [file pone.0203514.s001.docx]

Supplementary material

**Table A. Medication Use at Baseline and Three-Month Follow-Up**

**Table B. Temporal changes in Medication Use at Baseline and Three-Month Follow-Up**

| Medication Use | ACS | | | Non-ACS | | |
| --- | --- | --- | --- | --- | --- | --- |
|  | Enrollment | Follow up | p value | Enrollment | Follow up | p value |
| Aspirin | 28 (50) | 53 (95) | <0.001 | 52 (94) | 54 (98) | 0.32 |
| P2Y12 Inhibitor | 16 (29) | 44 (79) | <0.001 | 46 (84) | 52 (94) | 0.03 |
| Statin | 26 (46) | 49(87) | <0.001 | 47 (85) | 53 (96) | <0.001 |
| Beta Blocker | 28 (50) | 48 (85) | <0.001 | 46 (84) | 47 (85) | 0.57 |
| Calcium Channel Blocker | 6 (11) | 7 (12) | 0.67 | 25 (45) | 24 (44) | 0.32 |
| ACE Inhibitor | 17 (30) | 36(64) | <0.001 | 27 (49) | 28 (51) | 0.32 |
| ARB | 9 (16) | 6 (11) | 0.08 | 10 (18) | 8 (14) | 0.16 |
| Diuretic | 16 (29) | 15(27) | 0.71 | 19 (34) | 20 (36) | 0.57 |

Numbers presented as N (%)
