# Supplementary figures and images for: Systemic involvement in ACS: Using CMR imaging to compare the aortic wall in patients with and without acute coronary syndrome
Source: PLoS One. 2018 Dec 12;13(12):e0203514. doi: 10.1371/journal.pone.0203514 (PMC6291123; doi:10.1371/journal.pone.0203514)

***S1 Figure. Aortic wall area.***


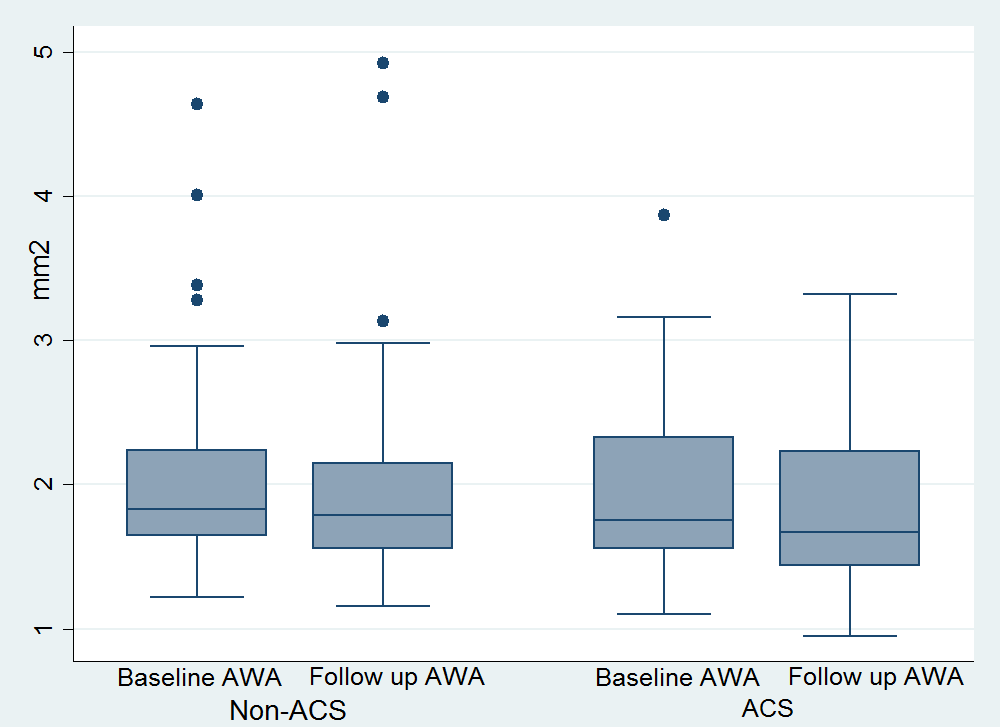


**AWA-aortic wall area**

**ACS- acute coronary syndrome**

Supplement: S1 Fig — (DOCX) [file pone.0203514.s002.docx]

***S2 Figure. Aortic wall thickness.***


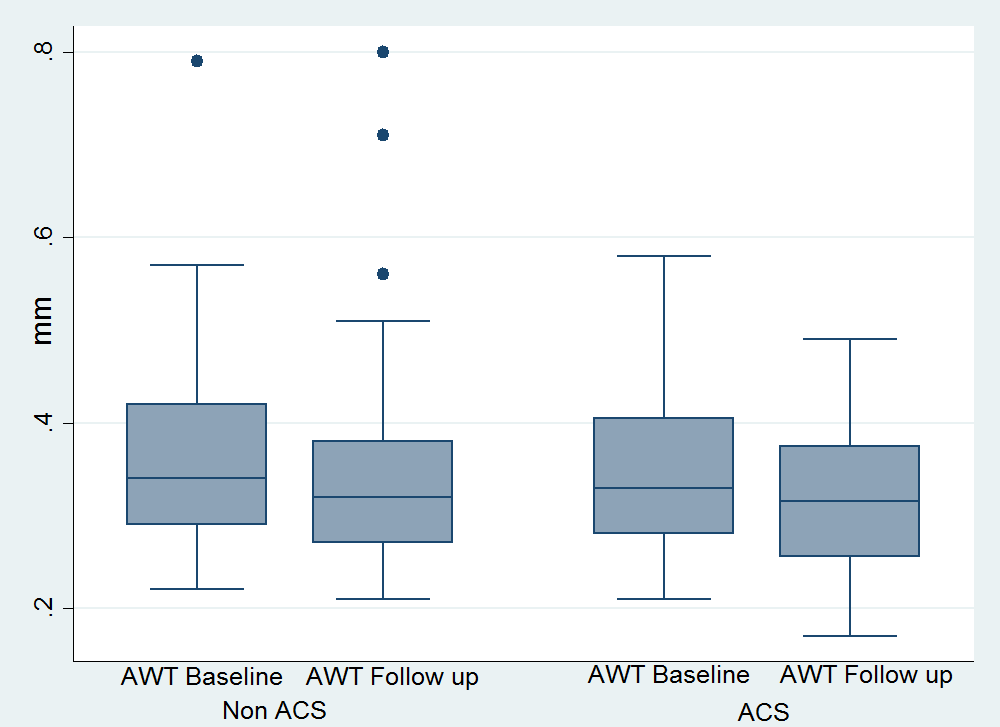


**AWT-aortic wall thickness**

**ACS- acute coronary syndrome**

Supplement: S2 Fig — (DOCX) [file pone.0203514.s003.docx]

***S3 Figure. Examples of CMR changes in Aortic wall area.***

***a)***


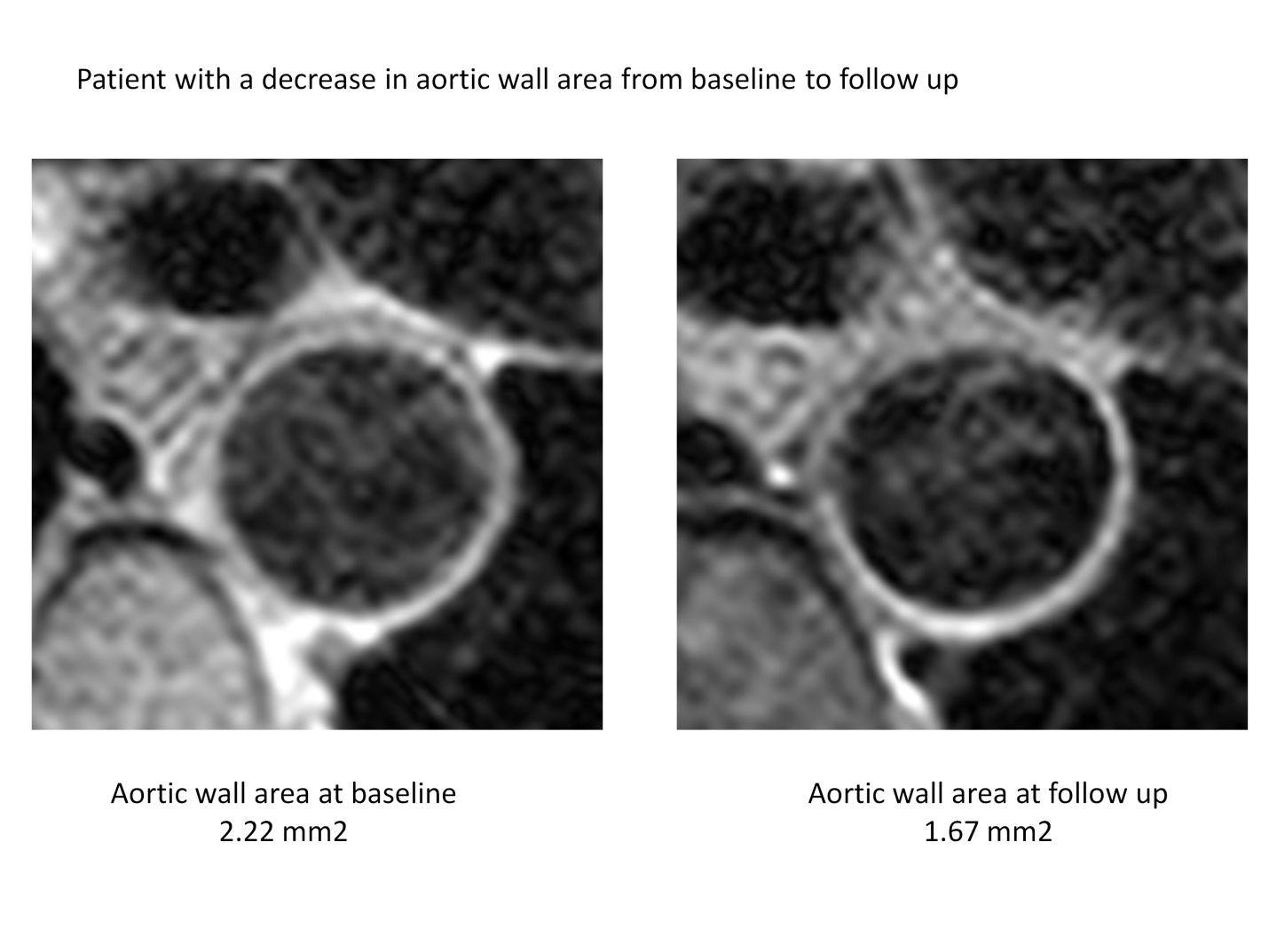


***b)***

***
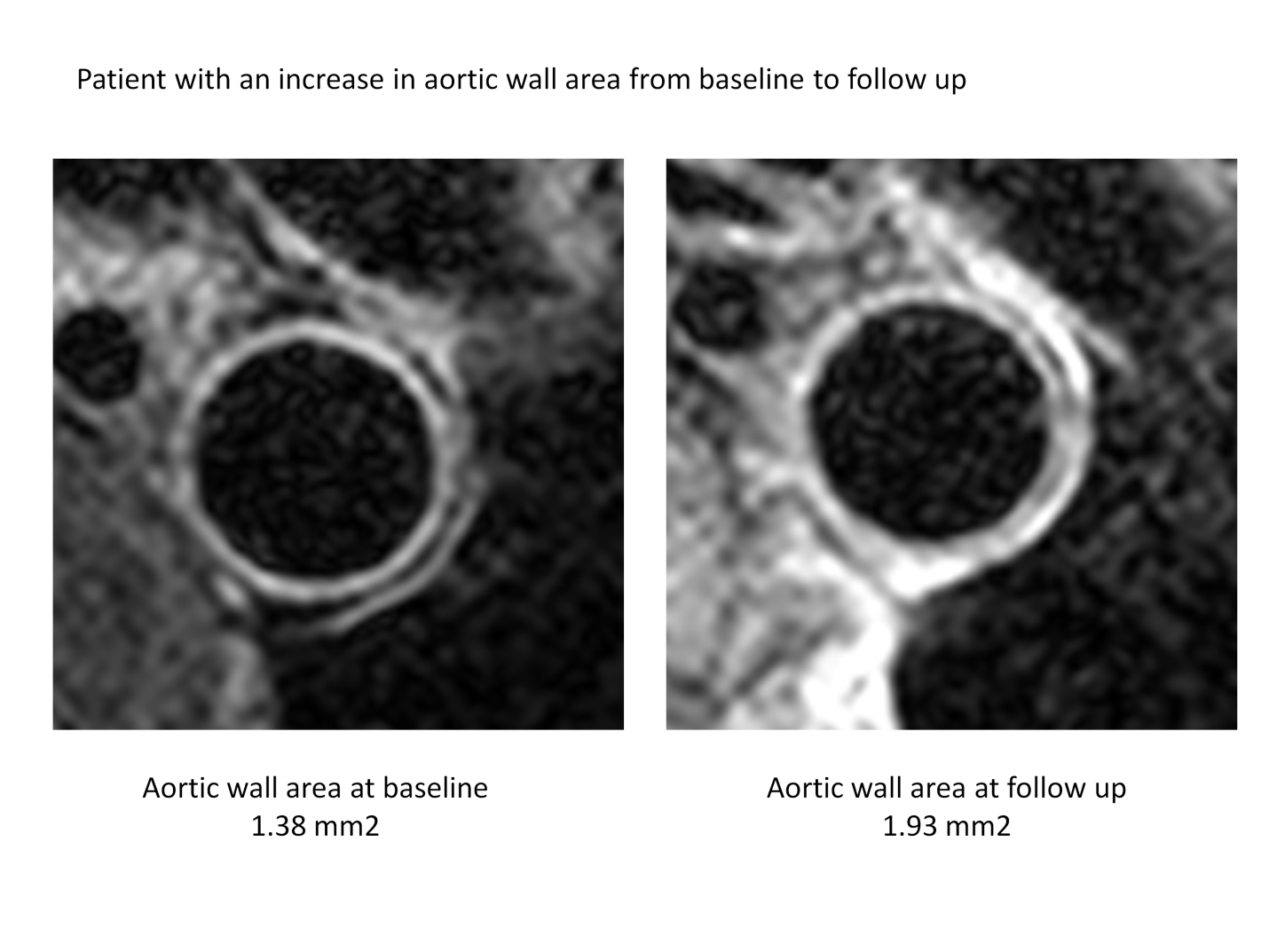
***

***c)
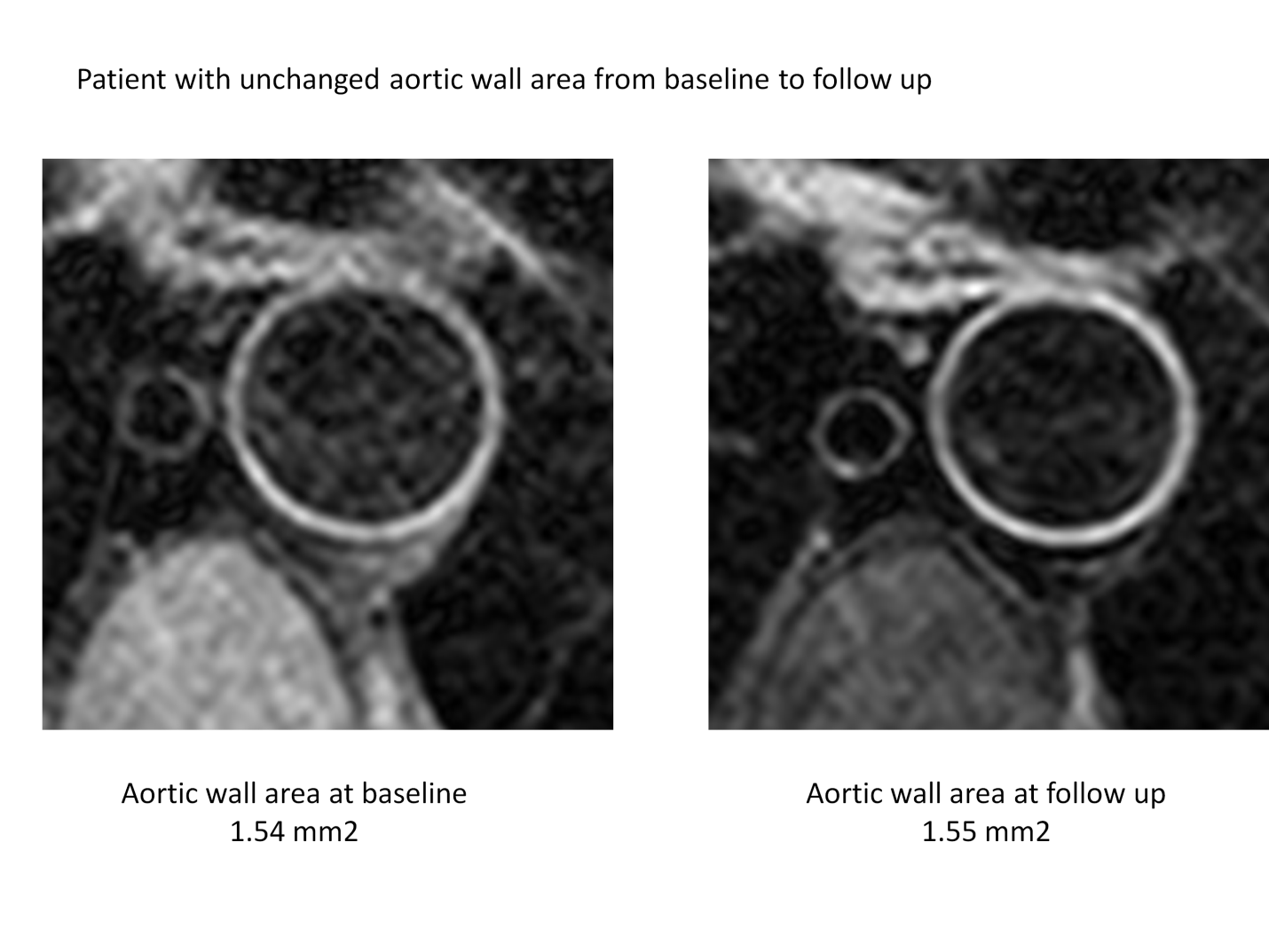
***

Supplement: S3 Fig — (DOCX) [file pone.0203514.s004.docx]
